# 

**Published:** 1889-03

**Authors:** 


					﻿Vol IX.
MARCH, 1889.
NO. 3.
ME
Homeopathic pn^iCiAjl,
A Monthly Journal of Homoeopathic Materia Medica
and Clinical Medicine.
“ He is a freeman whom truth makes free, and aU are slaves beside.”
“Seek the Truth; come whence it may, cost what it will.”
•	EDITED AND PUBLISHED BY
Edmund j.	m. ex,
AND
WALTER M. JAMES, M. D.
, PHILADELPHIA:
No. 1123 SPRUCE STREET.
z«Qxri>c> ir
ALFRED HEATH * CO., Sole Agents j?ok Great Britain,
114 Ebury Street, Eatou Square.
Yearly Subscription, $2.50. invariably in advance. Single numbers, 25 cts.
Bntwred at the Philadelphia Poet-Offlce aa Second-Claw Mail Matter.
				

